# Fungi Infecting Plants and Animals: Killers, Non-Killers, and Cell Death

**DOI:** 10.1371/journal.ppat.1003517

**Published:** 2013-08-29

**Authors:** Amir Sharon, Neta Shlezinger

**Affiliations:** Department of Molecular Biology and Ecology of Plants, Tel Aviv University, Tel Aviv, Israel; Duke University Medical Center, United States of America

## Similar Fungi, Different Hosts

Fungi occupy every inhabitable ecological niche on earth [1]. Environmental requirements vary, from species with very specific ones to species that can live under a broad range of conditions. Pathogenic fungi are those species that occupy and derive nutrients from living organisms. Some fungal pathogens completely depend on their host, while others can prosper in additional environments. Fungal host restrictions also vary considerably, from single-host-specific species to broad-host-range pathogens that can cause disease in a large number of different hosts. An extreme example is the genus *Fusarium*, with species that cause diseases in thousands of plant species as well as in animals, including humans [2]. Thus, while plants and animals present very different environments (hosts in the case of pathogens), the fungi that attack them are phylogenetically closely related. The same pathogenicity principles might therefore be used by animal and plant pathogens, albeit with some variation.

## Different Terminology, Common Strategies

Pathogens are often described by the nature of their relationship with their hosts. At one extreme are species that are entirely dependent on their host to complete their life cycle (often called obligate parasites). At the other are opportunistic species, which live as saprobes on dead organic matter, but can also invade living organisms (often called facultative pathogens). In between lies an array of combinations ranging in their degree of host dependency and ability to cause disease.

**Figure 1:**
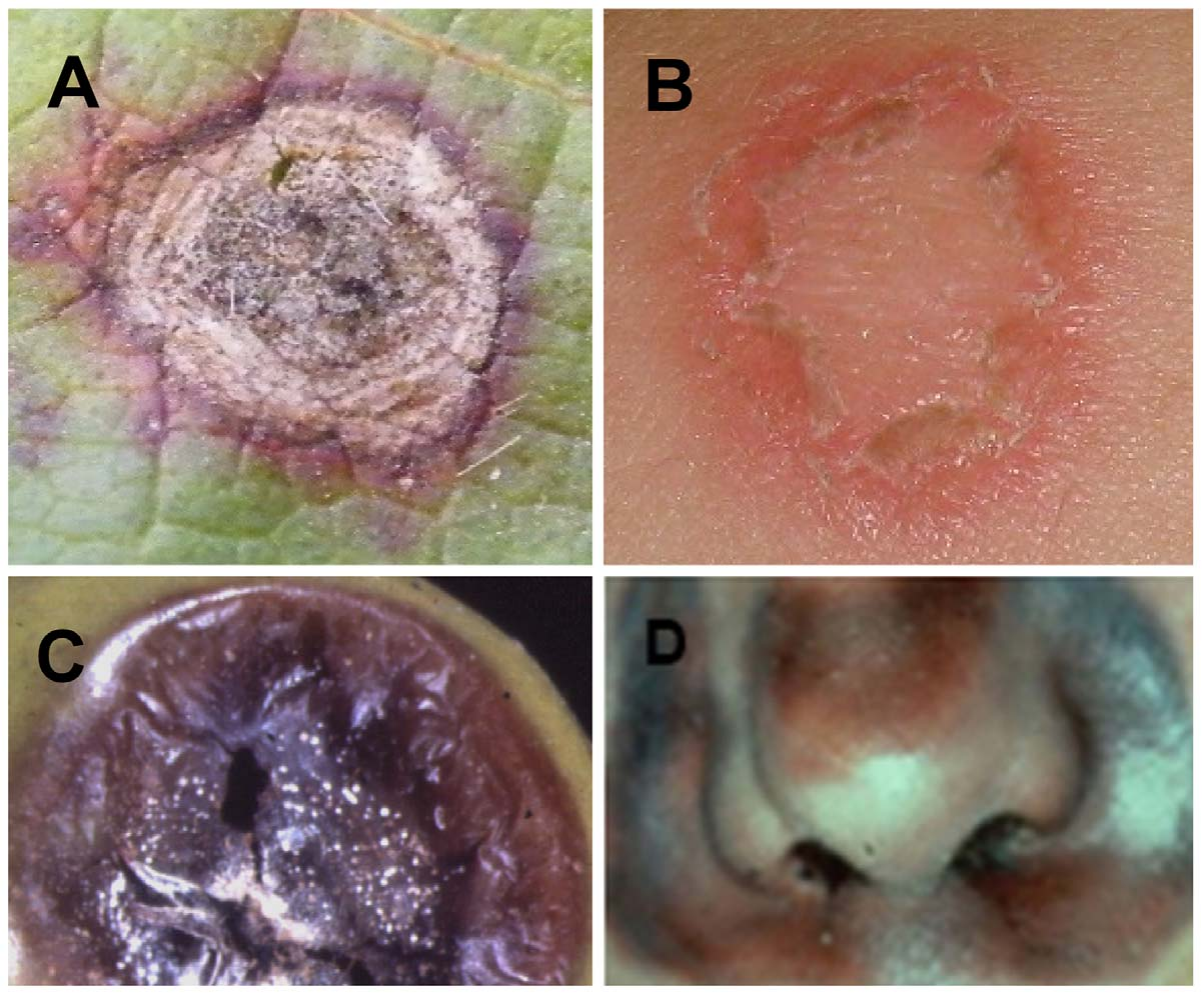
Similar symptoms caused by fungal pathogens on plants and humans. (A) Cercospora shot hole (plants), (B) dermatophytosis (ringworm) (human), (C) Botrytis rot (plant), (D) mucormycosis (human).

Another way to categorize pathogens is according to their pathogenic lifestyle and disease characteristics. In this case, different terminology is used for plant and animal pathogens: plant-attacking fungi are usually categorized according to the way they feed on the host, e.g., biotrophic or necrotrophic pathogens [3]. Fungi that cause disease in animals are usually described according to the type of disease they cause, e.g., superficial or invasive mycoses [4]. Therefore, we tend to think about fungal pathogens of plants and animals in different terms and treat them separately. Yet, as already noted, fungi attacking animals or plants are actually closely related. Moreover, close examination of animal and plant pathosystems reveals that fungal pathogens in both groups share similar infection strategies and sometimes even cause similar symptoms (although similarity in symptoms doesn't necessarily indicate similar mechanism) (Figure 1). For example, pH-lowering molecules, such as oxalic acid, are virulence factors against plant, animal, and insect hosts [5]–[7]. This warrants revisiting the terminology and the way in which we think about fungal pathogens of animals and plants.

## To Kill or Not to Kill: Toward a Unified Terminology

In the broadest sense, pathogens can be divided into **killers** and **non-killers**. The ultimate goal of killer pathogens is the conversion of living tissue into dead organic matter, and they are therefore programmed to efficiently kill host cells. Non-killer pathogens aim to keep their host alive, and are therefore programmed to prevent host cells from dying. In between there are a variety of intermediate situations, such as hemibiotrophs and dimorphic pathogens that switch their mode of action from non-killers to killers at different stages of their life cycle. Accordingly, host organisms use opposite defense strategies against killer and non-killer pathogens: they are programmed to activate self-cell death when facing non-killer pathogens, and block cell death when attacked by killer pathogens. Control of cell death is therefore a central element in all types of pathogen–host interactions. A major type of controlled cell death in animals is apoptosis, a type of programmed cell death (PCD) that is important for development as well as in pathogen interactions [8]. Apoptotic-like PCD is also known in plants, where it plays a role in defense [9], [10], and in fungi, where it is involved in pathogenic interactions with plants [11], [12]. Autophagy, a process that leads to recycling of intracellular organelles, is closely associated with apoptotic cell death and is part of the PCD-related processes that take place during pathogenic interactions [13].

Because plant and animal fungal pathogens are highly similar, involvement of PCD can be expected in all types of fungus–host interactions, including with animals.

## (Programmed) Cell Death: A Double-Edged Sword

If we accept the division of pathogens into killers and non-killers, it is easy to see that cell death must be handled very differently in each case. For example, if cell death is advantageous in defense reactions against one class of pathogens, it will ultimately have a counter effect against the other class. An example is the hypersensitive response (HR) of plants. The HR is a general mechanism that confers resistance against pathogens through a set of well-coordinated reactions that culminate in local death of cells at the site of pathogen invasion. While highly effective against non-killer pathogens, the HR is ineffective against killer pathogens [3]. In fact, killer pathogens such as the necrotrophic plant pathogens *Botrytis cinerea* and *Sclerotinia sclerotiorum* utilize the host HR to promote infection [14], [5]. Similar situations are known in animal pathogens [15]. For example, spores of the opportunistic human pathogen *Aspergillus fumigatus* enter the lungs. Under normal conditions, these spores are engulfed by macrophages that then undergo apoptosis, destroying them. Fungal spores that escape immune surveillance attempt to invade alveolar lung cells [16]. Studies have shown that the fungus produces different molecules, such as gliotoxin, that induce apoptosis of monocytes [17]. Similarly, mycotoxins produced by the human pathogen *Stachybotrys chartarum* activated apoptosis of alveolar macrophages [18]. In sharp contrast, non-killer pathogens use effectors that block the host PCD machinery [19]. For instance the intracellular fungus *Histoplasma capsulatum* induces antiapoptotic reaction in leucocytes as an escape mechanism [20]. Thus, the PCD machinery is targeted by both killer and non-killer pathogens, but it is manipulated in opposite ways. For example, the antiapoptotic protein BI-1 blocks PCD in plants. *BI-1* is necessary for successful infection of barley (*Hordeum vulgare* L.) by the powdery mildew fungus *Blumeria graminis*, a non-killer (biotrophic) pathogen [21], whereas overexpression of *BI-1* reduces plants' susceptibility to killer pathogens such as *Fusarium graminearum* and *B. cinerea* [22], [23]. These and other examples have made it clear that pathogens and hosts fight to gain control over the host PCD.

So far we have shown that host PCD plays a central role in all types of interactions with pathogenic fungi. Let us now examine this from the pathogen's side. An increasing number of reports show that PCD is the default response of fungi to harmful treatments and conditions, such as deleterious chemicals, antifungal drugs, or environmental stresses [24]–[26]. Furthermore, fungitoxic plant-defense compounds, such as phytoalexins and saponins, trigger fungal PCD [11], [26]–[28]. These data suggest that during their interaction with host cells, fungi are exposed to host-derived PCD-inducing molecules. This has been validated in the case of necrotrophic (killer) plant pathogens: the killer pathogens *B. cinerea* and *Cochliobolus heterostrophus* both undergo massive PCD during early stages of interaction with their host plants [29]. Furthermore, during the interaction between *B. cinerea* and *Arabidopsis thaliana*, fungal PCD is induced at least in part by camalexin, the main *A. thaliana* phytoalexin [29]. Genetic manipulation of the fungal anti-PCD response through knockdown or upregulation of the antiapoptotic protein BcBir1 reduced or enhanced fungal virulence, respectively. Thus, similar to the effect on plant susceptibility or resistance, manipulation of fungal PCD (by the host) can affect virulence.

To date, the connection between fungal PCD and virulence has only been demonstrated in plant-pathogenic fungi. However, in light of the similarities between fungal pathogens of plants and animals, and dividing all pathogens into killers and non-killers, we predicted that fungal PCD plays a role in all types of interactions, including that with animals. We therefore developed a system to study PCD in *A. fumigatus*, a killer-type pathogen of humans. Experiments using embryonated eggs demonstrated that *A. fumigatus* undergoes massive PCD soon after inoculation, and then fully recovers: a scenario that is highly similar to that observed in plant-necrotrophic fungi (our unpublished data). While preliminary, these results support the notion that fungal PCD is triggered not only during interaction of killer pathogens with plants, but also in other types of interactions, including those with humans.

**Figure 2:**
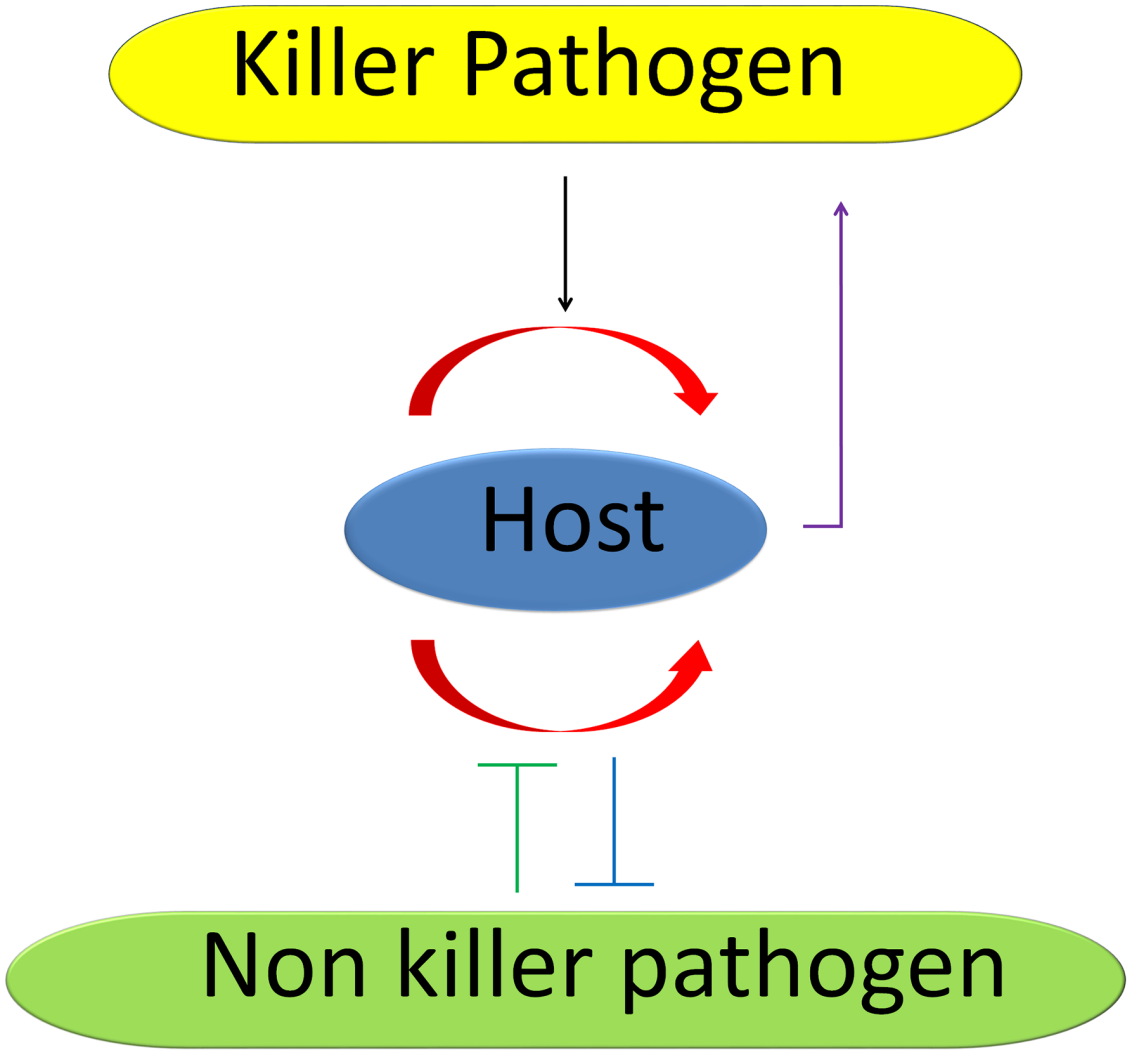
The “double-edged sword” model of cell death. Self-activated (programmed) cell death (**red** arrows) results in elimination of a small number of cells, effectively blocking the spread of “non-killer” pathogens (**blue** line). “Non-killer” pathogens use effectors to block host PCD as part of their infection strategy (**green** line). “Killer” pathogens thrive on this host response and use it to their advantage by activating PCD processes in the host (**black** arrow). Host organisms use a similar approach by targeting the fungal PCD (**purple** arrow).

Collectively, the data from animals and plants show that PCD plays multiple roles in all types of fungus–host interactions. It is also evident that PCD can be a double-edged sword: it is used to limit pathogen spread in certain types of interactions, but when controlled by the pathogen (e.g., by means of effectors), it can be turned against the host. A model of PCD-mediated fungus–host interactions is presented in Figure 2.

## Conclusions and Remarks

Fungal species that cause disease in plants and animals are closely related and therefore have evolved similar pathogenic strategies. We propose that all fungal pathogens can be collectively divided into killers and non-killers, a categorization that ultimately determines their infection strategy—immediate killing or prevention of death of host cells. As a consequence, cell-death-regulating pathways (collectively referred to as PCD) are central players in mediating hosts' defense against pathogens. Likewise, fungal PCD (in particular the anti-PCD machinery) is important for fungal virulence. Because PCD plays a central role in all types of fungus–host interactions, both the hosts' and pathogens' PCD machinery—and the pathways that regulate it—are putative targets for pathogen effectors and host defense molecules, respectively.
